# The value of domiciliary medication reviews: a thematic analysis of patient views

**DOI:** 10.1007/s11096-021-01288-1

**Published:** 2021-06-03

**Authors:** Patricia McCormick, Bridget Coleman, Ian Bates

**Affiliations:** 1 School of Pharmacy, University College London, 29-39 Brunswick Square, Bloomsbury, London, WC1N 1AX UK; 2 Pharmacy Department, Whittington Health, Magdala Avenue, London, N19 5NF UK

**Keywords:** Domiciliary, Medication review, Outcomes, Pharmacy practice, Stakeholder views, United Kingdom

## Abstract

*Background* Medication reviews are recognised as essential to tackling problematic polypharmacy. Domiciliary medication reviews (DMRs) have become more prevalent in recent years. They are proclaimed as being patient-centric but published literature mainly focuses on clinical outcomes. However, it is not known where the value of DMRs lies for patients who participate in them. *Objective* To determine the value of domiciliary medication reviews to service users. *Setting* Interviews took place with recipients of domiciliary medication reviews residing in the London boroughs of Islington and Haringey. *Method* Semi-structured interviews analysed using thematic analysis. *Main outcome measure* Themes and sub-themes identified from interview transcripts. *Results* Five themes were identified: advantages over traditional settings, attributes of the professional, adherence, levels of engagement and knowledge. *Conclusion* For many patients*,* the domiciliary setting is preferred to traditional healthcare settings. Patients appreciated the time spent with them during a DMR and felt listened to. Informal carers felt reassured that the individual medication needs of their relative had been reviewed by an expert.

**Supplementary Information:**

The online version contains supplementary material available at 10.1007/s11096-021-01288-1.

## Impacts on practice

- Individuals value DMR services. They like discussions in their own homes without the time constraints of traditional healthcare settings
- Individuals want to understand why they are being asked to take medications
- The connection with a professional during a DMR is extremely important for service users
- DMRs present an opportunity to address medication-related issues that other professionals do not know about

## Introduction

As the number of medications taken by an individual has risen [1] so too has the focus on medication reviews. Problematic polypharmacy has been shown to increase the likelihood of adverse drug reactions, drug interactions, medication-related hospital admissions, quality of life scores and the likelihood of non-adherence [2]. NICE recommends medication reviews to combat problematic polypharmacy as medication reviews have been shown to have a positive impact on these negative outcomes [3]. Medication reviews can occur in various settings and can have various levels of complexity [4]. It is comprehensive medication reviews, with shared-decision making (level 3) which are considered the gold standard for bringing about improvement in outcomes [5]. Domiciliary medication reviews (DMRs) are heralded as in-depth, comprehensive medication reviews, centred around the needs of the individual, and have become more prevalent in recent years. Previous studies have suggested that for patients, concern over medication related problems is a major motivating factor for participation DMRs [6, 7]. Published evidence around domiciliary medication reviews tends to focus on clinical outcomes, such as validated measures for medication appropriateness [8], suggesting this is where the value of these services lies. However, assessment of whether these services definitively have a positive effect on these outcomes varies [9–12]. There is a gap in the literature around where the value of these DMR services lies. It is not known whether the clinical outcomes reported are of value to the individuals who use the service or whether the value lies somewhere else. Given the proclaimed patient-centric nature of DMRs, these are important questions to answer.

### Aim of the study

The aim of this study was to determine the value of domiciliary medication reviews to service users through semi-structured interviews and thematic analysis.

### Ethics approval

The study was sponsored by University College London. Ethical approval was granted (18/NI/0049) by the Office for Research Ethics Committees Northern Ireland (ORECNI) in March 2018. Health Research Authority (HRA) approval (IRAS: 232128) was also granted before the study took place. Study participants were assured their identity and any information provided would be kept confidential. They were provided with written information on the study and formal consent was taken and documented before the interviews began. Participation was voluntary and subjects were free to withdraw at any time.

### Setting

Whittington Health is an Integrated Care Organisation (hospital setting) providing acute and community services to the London boroughs of Islington and Haringey. At the time of study Whittington Health had five professionals working across four DMR services. Potential research participants were identified from the DMR services provided by Whittington Health.

### Methods

Patients were recruited from DMR services provided by Whittington Health using convenience sampling. DMR pharmacists introduced the research to potential participants who met the following inclusion criteria:

1. More than 18 years old;
2. A recipient of a DMR;
3. Capacity to consent to participate in research;
4. Able to understand and communicate in English or has a family member/ carer that can translate for them.

They gave them an information leaflet and obtained their permission for their contact details to be passed to the researcher (PM). The researcher was a clinically-trained pharmacist with experience of carrying out DMRs. However, they were not engaged in patient care at the time of the study. The target sample size was 10–15 participants, or until data saturation was reached. The researcher telephoned potential participants, answered any questions they had and, if they were happy to participate in the research, arranged a convenient time for the interview. Formal consent was collected before the interviews began. If an individual requested, informal carers were also permitted to participate in the interviews. Interviews took place in patients’ homes. Semi-structured interviews were conducted using a topic guide. The topic guide focused on six areas:

1. The reason the DMR occurred, including from where the referral originated;
2. The individual’s expectations of the DMR;
3. The medication review;
4. The outcomes of the DMR;
5. The domiciliary setting;
6. The professional providing the service and any differences to interactions with other HCPs.

### Data collection and analysis

The target sample size was reached. Data collection took place between April and September 2018. Interviews were recorded and notes were taken during the interviews to aid data analysis. Audio files were transcribed into anonymised transcripts. Transcripts were checked against audio files to ensure transcription accuracy. Analysis of data followed a thematic analysis methodology [13]. NVivo® 11 software was used to manage the data. Analysis began through immersion in interview data. Each transcript was then coded. Codes were used to construct overarching themes from the data. Codes and themes were reviewed by both researchers (PM and BC) to ensure no duplication or ambiguity in meanings. Codes were compared and a consensus was reached through discussion. Both researchers felt that data saturation had been reached.

## Results

Twelve interviews were conducted. Eleven interviews involved one service user and one interview involved two: a husband and wife (P10 and P11). Three interviews also had an informal carer present (P1, P10 and P11, and P12). Seven (54%) of the participants were female and six (46%) were male. For the ten participants for whom demographic data were available, the average age was 84.4 years and the median 85.5 years. The average number of medications taken (n = 9) was 14.3 and the median 14.

Analysis of the semi-structured interviews revealed five key themes and 17 sub-themes (Table 1). Illustrative quotes are used to demonstrate the themes and sub-themes within transcripts.

**Table 1:** Themes and sub-themes from interviews

Theme 1: advantages over traditional settings	Theme 2: attributes of the professional	Theme 3: adherence	Theme 4: levels of engagement	Theme 5: knowledge
Mobility issues	Personability	Pill burden	Origins of DMR	Trusting the knowledge of acquaintances
Time spent	Competence	Side effects	Individual objectives	Medication expertise
Comprehensiveness	Accessibility	Independent decision to stop taking medication	Carer objectives	Importance of medication related information
Inter-professional differences			Shared decision making	

### Theme 1: Advantages over traditional settings

Individuals were asked their opinion on the domiciliary setting for professional interactions. A positive impact was reported in four key areas; the avoidance of encountering mobility issues, the amount of time the DMR pharmacist spent with individuals, the comprehensiveness of reviews and the positive patient-professional interaction during a DMR, presented in contrast to previous negative patient-professional interactions.

#### Mobility issues

Mobility issues were a barrier to accessing traditional healthcare settings, which were not encountered if a professional visited an individual in their home.

#### Time spent

Many participants recognised that the domiciliary setting enabled the pharmacist to spend more time with them. On occasion this was presented in contrast to the time spent with other healthcare professionals. Spending more time with a DMR pharmacist meant participants felt listened to, which corresponds to satisfaction with the service.

[Pharmacist name] has sorted it out. She was here for 1 hour and a half sorting it out. She wasn’t going to come in here for 2–3 min. She was here for 1hours and a half, 2 h and she sorted it all out [P9].

#### Comprehensiveness

The thoroughness and comprehensive nature of the DMR was highlighted on multiple occasions. It was recognised that the DMR pharmacist was trying to review the appropriateness of every medication taken. There were also examples of pharmacists picking up on, and trying to help resolve, non-medication related issues. The strongest recognition of the comprehensiveness came from informal carers.

She went through systematically each one and she said right we can cut that in half, we can do this and we can do that (–) she changed them [P9].

#### Interprofessional differences

Throughout the interviews the experience of a DMR was presented in contrast to participants’ previous experiences and interactions with other health care professionals, which they often found frustrating.

I come in, I’ve already told them exactly what’s the matter with me, I’m being sick, or whatever and then they ask me what I want. And then it’s 4 or 5 minutes on the computer looking back three hundred years ago what happened to me when I fell over… you know…by the time they do all that, they go oh, we’ll give you another two more pills. Because you know, you’re going to yourself, 5 minutes, you’ve only got 5 minutes to sort out what it is, and you might as well have not come. But they don’t understand that. They’re not doing that [P9]

There were also examples of other professionals ‘imposing’ interventions on participants without discussion, which resulted in confusion for the individuals.

For 15 years I've been taking stuff out of boxes, and all of a sudden, they come up with a blister pack and it doesn't mean a thing to me [P3]

### Theme 2: attributes of the professional

Three professional attributes were highlighted and discussed during the interviews: personability, competence and accessibility.

#### Personability

Several participants reported how ‘nice’ the professional carrying out the DMR was. Patients recalled the niceness of the professional more than they recalled their professional knowledge or capability.

I was glad to see him. He’s a nice boy [P5]

She’s a nice girl, I got on well with her, very nice person [P8]

#### Competence

The perceived professional competence of the individual was highlighted by two out of the three informal carers who participated in the interviews and only two out of the 13 service users.

He was very good, he looked through everything I got, chucked out a lot of it. and said you need this this and that… he’s very good [P4]

#### Accessibility

The opportunity to have a conversation with a health care professional was repeatedly highlighted. Service users felt heard and felt they could express their opinion.

I could speak to (pharmacist name) normal like I am speaking to you. Some people are not like that. [P2]

You know some people, you can talk to, and you can talk all day …to and some people you can look at (–) doctors, you know how they are and they’ve got this air about them and you don’t feel comfortable, all you want to do is get up and get out. Well, [Pharmacist name] you can sit and talk to for a week. [P9]

### Theme 3: Adherence

There were two issues that affected medication adherence in the sample of individuals who participated in the interviews: the pill burden and side effects.

#### Pill burden

Pill burden was highlighted in several interviews, in a negative context, linked to non-adherence.

It can get you down [taking medications everyday] I mean this did get me down at first but I've got used to it. [P2]

#### Side effects

Participants reported that side effects were a reason for them to stop taking their medications. Reported side effects included constipation, how they make a person feel e.g. not in control, excessive sleepiness, weight gain and gastrointestinal problems.

#### Independent decision to stop taking a medication

During the interviews there are examples of participants deciding not to take a medication they had been given as they felt they did not need to take it.

I had a fall they gave me some morphine but I didn’t take them, I just stuck to my paracetamols. [P8]

I never used it [inhaler] so I’ll have to take it back. [P13]

### Theme 4: Levels of engagement

There were differences in how DMRs came about, how involved individuals wanted to be with the DMR process and the degree of shared decision making.

#### Initiation of the DMR

11 out of 13 participants did not request their DMR. Of the DMRs that were requested by the service user, the first was by the participant’s (P9) wife because of worries about polypharmacy. The second was opportunistic; the participant (P11) requested the review while the DMR pharmacist was in his home to review his wife’s (P10) medications.

#### Individual objectives

Although most reviews were not requested by individuals, six participants wanted to discuss a medication related issue when they became aware of the DMR. P3 wanted the excess medications in their home to be removed. P6 and P7 wanted to know whether their medications were beneficial. P13 wanted a bigger tablet box and to discuss one of their inhalers. P7 did not request the DMR but they did actively approach their GP to discuss their medications:

I was worried I was taking too many; I was worried that some of the tablets might be clashing. I’ve got so many things wrong, I’ve got about 14 different conditions. And I wanted to know if the pills were right and I wanted a review. [P7]

In one DMR a participant did not engage with the DMR process because she was prioritising the needs of her husband. Instead, her informal carer took a lead role.

I suppose I was more interested in what was playing out for my husband…It wasn't really worrying me. I was taking medication and getting around and looking after my husband. [P1]

#### Carer objectives

Informal carers wanted assurance that the medications their family members were being asked to take were appropriate. They also wanted to increase their understanding around medication indications.

It was just making sure that the interplay of the medication that she was having. You know, she wasn't taking one thing to the detriment of something else. That was another really important reason for having that reviewed. [Informal carer of P1]

#### Shared decision making

During interviews participants gave examples of them making suggestions around changes to their medications, but no one recalled a true shared decision-making experience.

They cut it in half. I suggested that. See, sometimes you can suggest it [P2]

But we cut them down. I said listen, I can’t not go without them. But I don’t need one in the morning and one at night. I only need one. [P9]

### Theme 5: Knowledge

Knowledge is a key theme from the interviews, particularly who provides this knowledge. Interviewees take knowledge from professionals and acquaintances. No individual presented themselves as the definitive source of knowledge. Once medication related knowledge was acquired, it was of importance to the individual.

#### Trusting the knowledge of acquaintances

P1 took the advice of an acquaintance and stopped taking a medication because of a potential side effect. Avoiding the side effect was more important than treating their pain.

The only thing I found out about today or yesterday rather that someone else who has had a similar sort of thing, is that codeine give you constipation…. It just that she (visitor) said I've noticed it since taking them and I thought oh that's funny. [P1]

#### Medication expertise

During the interviews there were examples of participants taking the advice and information provided the DMR pharmacist:

There was a conversation about the importance of actually taking paracetamol and codeine together to have effective pain relief [carer of P1]

Another participant recalled their GP stating they were not a medication expert, suggesting that not every professional can provide medication expertise.

Well my doctor really. And I’ve got a lot of time for him. And he said “look [interviewee name], I’m not qualified to do this, I’m a GP, not an expert on tablets. You’re probably right but I don’t know which ones might clash and which ones are wrong. And yes, I do it piecemeal but that’s the only way I can do it”. [P7]

#### Importance of medication related information

When medication information has been given to participants by the DMR pharmacist there were references to it making a difference to the individual or resulting in a change of medication taking behaviours.

Until this came [refers to medication reminder chart given by DMR pharmacist], I hadn't got a clue [P3]

## Discussion

Removing the need to travel to a traditional care setting—such as a hospital or clinic—was a strong positive impact for patients for whom leaving the house is a challenge. During the DMR there was more of an opportunity to show and discuss medications. It is known that DMRs uncover access, adherence and clinical, drug related problems [14]. This study suggests DMRs also uncover non-medication related issues. The comprehensive nature of DMRs was valued, particularly by carers. Being an informal carer can put psychological strain on the carer and they can feel they are operating without support [15, 16]. A comprehensive DMR removed confusion and stress around appropriateness of medication therapies. The ability to pick up on, and help resolve, non-medication related issues was also appreciated.

The time spent in conducting a DMR was valued. Earlier work conducted by the researcher found that DMRs take an average of 46 min (± 19) min [17]. Inter-professional differences were highlighted in the context of lack of time, particularly for GPs. Descriptions of these interactions left individuals feeling frustrated and misunderstood. In the UK a GP consultation will last for an average of 9.2 min [18] It is widely known that GP services are under pressure, and the feeling of frustration is echoed by GPs themselves; who worry about the quality of care they can provide [19]. If an in-depth consultation around medications, which requires time, can be carried out by a pharmacist with expertise in the area this could avoid negative patient-professional interactions.

Inter-professional differences were also highlighted through descriptions of blister packs being imposed on a participant when they did not feel they needed one. Inappropriate blister pack use has been highlighted as problematic by the Royal Pharmaceutical Society (RPS), but they have been recommended and by a range of professionals as a one size fits all approach to adherence which does not address the underlying issues [20]. Memories of decisions that individuals did not agree with or consent to stayed with them. These experiences were presented in contrast to the DMR encounters where there was an attempt to consider individual wishes.

The personability of the pharmacist, particularly their ‘niceness’ and ability to engage was valued by participants. The personability of the professional correlated to individuals feeling the professional was accessible. Accessibility gave participants a way to raise further questions, which was appreciated. The authors of the HOMER trial [21] looked at the attributes of the pharmacists conducting DMRs to see if this made a difference to outcomes, but the focus was on professional attributes and experience level not the ‘softer’ attributes that interviewees highlighted as important. DMR service managers should examine ways to skill DMR pharmacists, enabling them to engage individuals in conversations about their medications and wider needs. The competence of the pharmacist was valued by the informal carers who participated in the interviews.

Pill burden and side effects are known reasons that individuals become fatigued with medication taking [22]. DMR interventions that address pill burden and medication related side effects are important to individuals, and therefore the value they see in the DMR service. If an individual has taken an independent decision to stop a medication the DMR presents an opportunity to discuss this decision. Without the DMR the non-adherence may not have been picked up another professional. DMRs also overcome the barriers individuals face when they want to discuss their concerns; access to an HCP and lack of time allocated to consultations when they do access one.

Most of the participants did not request a DMR; it was suggested by a professional. Despite not requesting the review no participant described themselves as irritated that the DMR took place. The interviews revealed instances of individuals wanting to be involved with decisions about their medications. Although, there were no examples describing true shared decision making. DMR pharmacists should be trained in ensuring they are having two-way conversations with individuals. They should explain their intentions fully and be able to detect when a person does agree with the reason the DMR is taking place and respond appropriately. Shared decision making is a national priority [23], but it may not be a priority for the individual. Previous work examining the information needs of hospital patients proposed that “a desire for information is not the same as shared decision making” [24]. A systematic review conducted by Willeboardse et al. [25] examining healthcare professional and patient interactions concluded they rarely go beyond information exchange. If this is a phenomenon we are also observing within DMRs we need to examine why.

For the three informal carers expressed a desire to understand the medication their relatives were being asked to take and looked for reassurance that the medications were appropriate. The objectives of the informal carers were more aligned to the traditional skill sets of pharmacists i.e. providers of medication expertise. There appears to be value in including informal carers in the DMR process.

Patients obtain healthcare knowledge from a range of professional and supplemental sources [26]. In this interview sample there were examples of the DMR pharmacist providing information to the individual which had an impact on medication taking behaviours. There is a suggestion that the provision of medication information can add value. The one example of a GP presenting themselves as not an expert in medications raises and interesting discussion point around which professionals should be conducting in-depth, complex medication reviews. One of the participants trusted the knowledge of an acquaintance enough to alter her medication taking behaviour. DMR pharmacists need to have the skills to assess the validity of the information that individuals have taken on, and perhaps challenge it in a respectful way, while maintaining trust so that the individual recognises and accepts their expertise.

Limitations of the study include interviews involved a relatively small sample size. However, data saturation was reached and consistent themes were identified. Another potential limitation is selection bias. Participants were initially recruited by the pharmacist who conducted the DMR. There is potential that they selected patients who they felt would report favourable outcomes from the DMRs. This was countered by asking a range of questions to capture views on multiple aspects of the DMR. A third limitation is that participants were recruited from two boroughs in London; findings may not represent those of DMR participants nationally.

## Conclusion

To our knowledge this is the first in-depth exploration of the opinions of DMR service users. Where the value lies for the people who use the service has not been explored or presented in the literature. The value of DMRs is afforded through the domiciliary setting and the time spent, permitting longer in-depth interactions between individuals and the DMR pharmacist. This has important implications for service provision, pharmacists need to be afforded enough time to have comprehensive conversations. The emphasis should be on quality of the DMR interaction, not the quantity of DMRs completed. Positive experiences of the DMR were linked to the personability of the pharmacist and their ability to engage. The lasting impact of the DMR was that individuals felt listened to. Informal carers valued professional knowledge and felt reassured that someone had taken the time to ensure medications taken were appropriate for their family member. Future work should examine whether these findings are mirrored in a larger sample of informal carers. There is a mismatch between where DMR service users (patients and carers) feel the value of the service lies and the frequently reported clinical outcomes in the literature. Future work around DMRs should examine ways to capture outcomes that are important to the people who use these services.

## Supplementary Information

Below is the link to the electronic supplementary material.

Supplementary file1 (DOCX 22 KB)x
