# Supplementary material for: The value of domiciliary medication reviews: a thematic analysis of patient views
Source: Int J Clin Pharm. 2021 Jun 3;43(6):1594–601. doi: 10.1007/s11096-021-01288-1 (PMC8642367; doi:10.1007/s11096-021-01288-1)
Supplement: Supplementary file 1 — Supplementary file1 (DOCX 22 KB)x [file 11096_2021_1288_MOESM1_ESM.docx]

**Topic guide: Service user stakeholder opinion on the value of domiciliary medication reviews**

*Please note: this topic guide is evolving document. Questions may be added based on findings of previous interviews. All questions will be related to aims and objectives described in protocol.*

**Introduction:** As I mentioned on the telephone, my name is Patricia McCormick. I am carrying out research looking into the experiences of people who have had medication reviews in their home as part of my PhD. I would like to ask you some questions about your recent medication review. I will record our conversation so that I have a record of our conversation for analysis purposes. I will also make some notes. Please do not be put off by this.

Any questions before we begin?

Are you happy to start?

PART 1: How did the DMR come about

Somebody visited you recently to talk to you about your medicines. Tell me how this came about

*(the interviewer will need to have a brief understanding of which service and individual provided the review for context)*

**Prompting questions:**

1. did you request the review?
2. request information on who requested or suggested review if not service user

PART 2: Expectations of DMR

(*If not answered during response to first question)* Did you have specific issues that you wanted to resolve during the medication review?

**Prompting questions:**

1. Can you tell me what the purpose of the review was?
2. What did you want to get out of the review?

PART 3: The DMR

Tell me what happened during your DMR

**Prompting questions:**

1. What type of things were discussed?

Part 4: Outcomes of DMR

What changed because of the medication review?

**Prompting questions:**

1. did anything change with regards to your medications after the review?
2. *Potential examples which can be given wo help stimulate conversation: some people have medications stopped or started when they have medication reviews or they may have been given information to help them understand what their medications are for*
3. if yes, ask whether the changes make a difference to your everyday life?
4. if no, clarify: No changes were made to your medications as a result of the medication review?
5. why do you think changes were made?/ Why do you think changes were not made?
6. How much did you know about your medications before the medication review?

PART 4: The setting

How did you find having a medication review in your home?

**Prompting questions:**

1. have you had a medication review before in another setting*, give examples if necessary e.g. in your community pharmacy, at your GP surgery*
2. How was this medication review different? *Probes:* Was anything better? OR Was anything worse?
3. when presented with differences ask whether these issues are important to the interviewee?
4. In general, how do you feel about the number of medications you are prescribed to take?
   1. Do you mind having to take medications
   2. Do you feel you take too little or too many?

PART 5: The professional providing the service

Can you tell me a bit about the person who can to your home to carry out the medication review?

**Prompting questions:**

1. did you feel comfortable talking to them?
2. did they explain who they were and what they did?
3. did they explain how the review would take place?
4. ‘What did you particularly like / the best thing about the pharmacist who came to visit you?’
5. Are there any differences between this person and other pharmacists you have had an interaction with? What are these differences?
6. In general, do you like to be involved with decisions about your health OR are you you happy for professionals to make decisions on your behalf
7. Could the pharmacist have done anything differently?

PART 6: Finally

Would you like to ask me any questions?

Examples of general probes:

And then what happened?

Can you tell me more about that?

Anything else?
